# Food-Derived Bioactive Molecules from Mediterranean Diet: Nanotechnological Approaches and Waste Valorization as Strategies to Improve Human Wellness

**DOI:** 10.3390/polym14091726

**Published:** 2022-04-23

**Authors:** Ilenia De Luca, Francesca Di Cristo, Anna Valentino, Gianfranco Peluso, Anna Di Salle, Anna Calarco

**Affiliations:** 1 Research Institute on Terrestrial Ecosystems (IRET)—CNR, Via Pietro Castellino 111, 80131 Naples, Italy; ilenia.deluca@iret.cnr.it (I.D.L.); anna.valentino@iret.cnr.it (A.V.); gianfranco.pelso@unicamillus.org (G.P.); anna.calarco@cnr.it (A.C.); 2 Elleva Pharma s.r.l. via P. Castellino, 111, 80131 Naples, Italy; francesca.dicristo@ellevapharma.com; 3 Faculty of Medicine and Surgery, Saint Camillus International University of Health Sciences, Via di Sant’Alessandro 8, 00131 Rome, Italy

**Keywords:** polyphenols, Mediterranean diet, delivery system, nanotechnology

## Abstract

The beneficial effects of the Mediterranean diet (MedDiet), the most widely followed healthy diet in the world, are principally due to the presence in the foods of secondary metabolites, mainly polyphenols, whose healthy characteristics are widely recognized. However, one of the biggest problems associated with the consumption of polyphenols as nutraceutical adjuvant concerns their bioavailability. During the last decades, different nanotechnological approaches have been developed to enhance polyphenol bioavailability, avoiding the metabolic modifications that lead to low absorption, and improving their retention time inside the organisms. This review focuses on the most recent findings regarding the encapsulation and delivery of the bioactive molecules present in the foods daily consumed in the MedDiet such as olive oil, wine, nuts, spice, and herbs. In addition, the possibility of recovering the polyphenols from food waste was also explored, taking into account the increased market demand of functional foods and the necessity to obtain valuable biomolecules at low cost and in high quantity. This circular economy strategy, therefore, represents an excellent approach to respond to both the growing demand of consumers for the maintenance of human wellness and the economic and ecological exigencies of our society.

## 1. Introduction: The Mediterranean Diet as a Healthy Lifestyle

The Mediterranean diet (MedDiet) is one of the healthiest dietary patterns and is characterized by a high and balanced consumption of fruits and vegetables, nuts, cereals, fiber, and olive oil [1,2,3]. Although the expression “Mediterranean diet” refers to the dietary model followed by people living around the Mediterranean Sea, the dietary habits are very heterogeneous with few common foods such as fruit, vegetables, olive oil, fish, and wine. Indeed, the MedDiet is the fruit of centuries of eating habits based on agricultural and rural traditions of each region, so it is quite heterogeneous not only among the countries of the Mediterranean Basin but also within the countries themselves. Moreover, the food pyramid, first developed by the Oldways Preservation Trust, the Harvard School of Public Health, and the World Health Organization in 1993 and characterizes the eating pattern of the MedDiet is constantly evolving to obtain a common representation of the health, nutritional, sociological, and environmental heritage of the Mediterranean area [4,5]. The latest update held during the MedDiet conference organized by CIHEAM-Bari and the Forum on Mediterranean Food Cultures in Palermo in May 2019 involves the daily intake of olive oil as a principal source of dietary lipid; of nuts and seeds in a reasonable quantity (i.e., a handful) as a healthy snack; of herbs, spices, garlic, and onions to give dishes flavor, consenting to a reduction in salt use [4].

Already in 2010, UNESCO recognized the MedDiet as an intangible cultural heritage of humanity, while in 2016, the United States Department of Agriculture recommended the adherence to this dietary model in the Dietary Guidelines for Americans 2015–2020 [6]. The World Health Organization (WHO) estimated that the risk associated with cancer death could be reduced by modifying eating habits, lifestyle, physical inactivity, smoking, and alcohol consumption [7]. Recently, a group of researchers estimated that dietary risks affected people regardless of age, sex, and sociodemographic development of their place of residence and that an improvement of diet could potentially prevent one in every five deaths globally [8]. In this context, during the last century, the MedDiet model has gained importance, becoming an inestimable instrument not only to avoid the onset of numerous diseases but also to contribute to well-being.

The European pioneer large nutritional trial “Prevención con Dieta Mediterránea” (PREDIMED) Study determined that the adhesion to the Mediterranean diet supplemented with extra-virgin olive oil (EVOO) or nuts with respect to a reduced-fat diet, reduced by approximately 30% the incidence of major cardiovascular events [6]. In particular, the study demonstrated that EVOO, when administered approximately 1 L per week, was able to induce a higher effect [Hazard Ratio (HR):0.69 (95% confidence interval, 0.53 to 0.91)], reducing the onset of a cardiovascular event such as myocardial infarction, stroke, or death from cardiovascular causes. Moreover, a large Spanish prospective study showed that higher adherence to a MedDiet was inversely associated with the incidence of type 2 diabetes among initially healthy participants [9]. The same results were obtained in the European Prospective Investigation into Cancer and Nutrition study (EPIC) [10], and in a very recent U.S. study [11]. The Italian GISSI-Prevenzione trial including 8291 patients with a recent myocardial infarction demonstrated that the MedDiet protected against new diabetes (35% lower risk) [12].

The beneficial effects of the MedDiet on cardiovascular diseases, metabolic syndrome, cancer type II diabetes, allergy, and neurological diseases is mainly due to the high concentration and synergistic effect of the phytochemical components of these foods such as polyphenols, sterols, peptides, polyunsaturated fatty acids, vitamins, etc. [13]. In general, phytochemicals are synthesized in plants as defense agents against various insults including infections, environmental, and nutritional factors [14]. These compounds, and in particular polyphenols, have gained more attention during the last years due to their health effect against certain types of cancers, cardiovascular diseases, metabolic syndrome, and neurodegenerative diseases [15,16,17,18].

Commonly, the biological properties of polyphenols are attributed to their antioxidant activity according to the “biochemical scavenger theory” [19] or in maintaining cellular redox state homeostasis [20]. Moreover, several different molecular mechanisms have been described including the modulation of intra- and inter-cellular signaling pathways such as the activation of antioxidant enzymes [21], the regulation of lipid metabolism [22], the activation of nuclear transcription factors [23], the modulation of the inflammatory pathway [24], or key cancer processes such as proliferation, apoptosis, and differentiation [25]. In addition, recently, it was highlighted that plant-derived phytochemicals such as resveratrol, quercetin, epigallocatechin-3-gallate, and curcumin modulate and interfere in bone tissue regeneration [26].

Furthermore, dietary polyphenols may influence epigenetic mechanisms of aging-related diseases such as cancer, lung disease, nephrosclerosis, cardiovascular disease, diabetes, and arthritis. Indeed, Arora et al. highlighted that diet polyphenols as quercetin, curcumin, resveratrol, and apigenin were able to modulate multiple signaling pathways such as DNA methylation, DNA demethylation, histone modifications, and gene expression [27].

Again, the molecular effect of polyphenols was also demonstrated for DNA damage in different types of cancer. In fact, polyphenols could target DNA damage response sensors, transducers and mediators, affecting apoptosis last [28].

**Figure 1:**
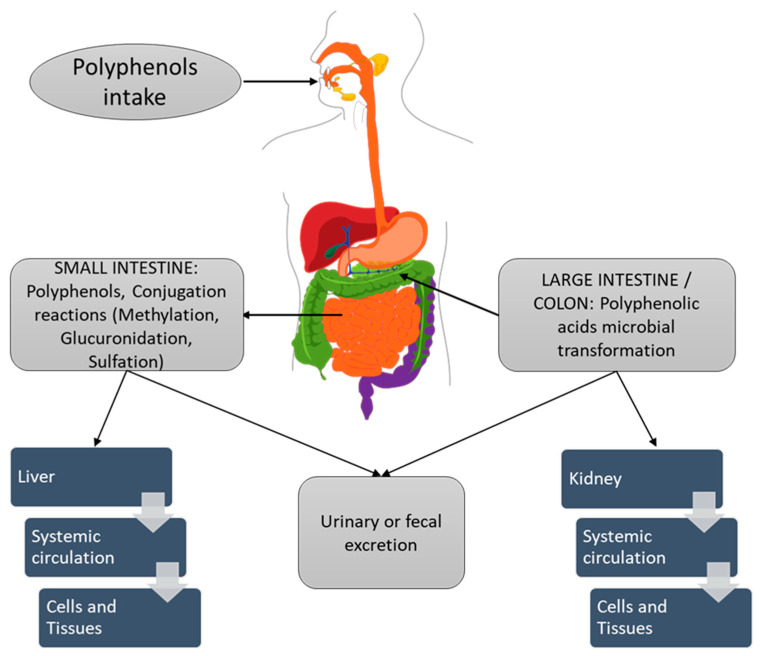
Schematic representation of polyphenol absorption after intake.

However, EPIC and PREDIMED studies [6,10] have shown that large quantities of polyphenol-rich foods must be taken with the diet to have a visible effect on health. Indeed, the polyphenol bioavailability is very low due to the lower absorption from the intestine, the extensive gut transformation, and the rapid clearance from the body. Once ingested, polyphenols, similar to many xenobiotics, undergo several transformation processes such as glycosylation, methylation, glucuronidation, and sulfation, which reduce their potential toxicity, limiting their diffusion across barriers in the gastrointestinal tract and facilitating their biliary and urinary elimination by increasing their hydrophilicity (Figure 1) [29]. Several polyphenols available as glycosylated compounds present a low diffusion across barriers in the gastrointestinal tract. Polyphenols are extensively transformed via phase II pathways, predominately methylation, glucuronidation, and sulfation in the enterocytes of the small intestine, and then further metabolized in the liver, facilitating their quick excretion. Additionally, polyphenols are secreted via the biliary route into the duodenum, where they are transformed by bacterial enzymes such as glucuronidase, and reabsorbed in cells and tissues or excreted.

In addition, the clinical relevance of the administration of polyphenols as pure principles may also be reduced for the possible interactions with other diet molecules [30]. On the other hand, the poor bioavailability allows dietary polyphenols to reach the colon in unaltered form, where microbiota can metabolize them into bioactive secondary compounds, many of which have anti-inflammatory activity and higher bioavailability than their precursors [31]. For example, ellagitannins and ellagic acid are transformed by intestinal microbiota into anti-estrogenic and anticancer compound urolithins that are much better absorbed [32]. Moreover, recent scientific evidence has shown that dietary polyphenols acted as prebiotics, enriching the microbiota composition in beneficial bacteria, in particular, *Bifidobacteriaceae* and *Lactobacillaceae* [33].

Several strategies have been designed to increase the chemical stability or bioavailability of food polyphenols. Indeed, most food-derived bioactive compounds such as polyphenols are water-insoluble, resulting in poor bioavailability and little effectiveness for health.

Traditionally, the approaches involved the addition of reducing agents to preserve the chemical structure, the use of dissolving agents to increase solubility, the addition of lipids or protein-like albumin, or the modification of polyphenol structure with the addition of glycosyl groups [15]. Another strategy includes the modulation of phase I and II enzymes involved in polyphenol metabolism [34]. However, these strategies could induce an increase in adverse effects such as gastrointestinal inflammation [35].

Nevertheless, polyphenols are often susceptible to oxidative deterioration, leading to unpleasant flavor taste, thus affecting the consumers’ compliance [36]. Among the strategies to overcome these hindrances, the encapsulation in delivery systems could represent the better strategy [37]. In this context, nanotechnology, with the polyphenol encapsulation in nanovectors (i.e., matrix systems, solid dispersions, and liposomes), provides new instruments to improve polyphenol delivery, distribution, bioactivity, and consumer compliance [15,38].

Principally, nanotechnology was developed to enhance local bioavailability or drug delivery to a specific target tissue, avoiding the metabolic modifications that could lead to low absorption, thus improving the retention time of bioactive molecules inside organisms [39,40].

Different nanostructured encapsulation systems have been optimized for bioactive molecule delivery: liposomes, nanoemulsions, microemulsions, solid lipid nanoparticles (SLNs), and polymeric nanoparticles (Table 1).

In the biomedical field, these vehicles can be dispensed as oral, intravenous, intraperitoneal, and transdermal administration according to the target site and the goal to reach [2]. In detail, to improve the efficiency and bioavailability of phenolic molecules, lipid-based nanocarriers are commonly used for biomedical applications. Due to their lipidic composition, these carriers can promote the transition of polar phenolics from aqueous parts to the bloodstream. Interestingly, this system can also be used in beverages and aqueous foods, since they do not affect the taste of products [73].

This review focused on the most recent findings relating to the encapsulation of the bioactive molecules characterizing the foods of the MedDiet such as olive oil, wine, nuts, spice, and herbs, which are consumed daily. A particular mention will be made about resveratrol, a polyphenol contained in very high percentages in wine whose healthy characteristics are widely known. The MedDiet, while fully respecting religious ideologies and social rules, contemplates moderate consumption, preferably during meals, of wine.

Moreover, particular attention is paid to the polyphenol’s recovery from food waste to respond both to the economic and ecological exigencies of our society [74].

## 2. Olive Oil

Olive oil, obtained from olives (*Olea europaea*, family Oleaceae), is the most important and common ingredient to all the countries of the Mediterranean Basin and is used as the main dietary source of fat. As established by the European Council Regulation (EEC) No. 356/92, the oil produced with a process that preserves all bioactive molecules by using only the mechanical pressing of olives, without any thermal or chemical treatment following, is called virgin olive oil when the free acidity is up to 2.0%, or extra virgin olive oil when the free acidity is lower than 0.8%.

Extra virgin olive oil (EVOO) shows numerous nutraceutical properties due to its bioactive components (Table 2) [75].

Traditionally, the beneficial effects of EVOO have been ascribed to its high content of MUFAs, particularly oleic acid, which has shown multiple beneficial properties [76,77]. Indeed, the oleic acid (C18:1 *n*-9) concentration is about 55–83% higher than that of the other fatty acids (linoleic, palmitic, or stearic acids, ranging between 3% and 21%). However, in recent years, converging evidence (in vitro and in vivo studies, human, and animal) indicates that the EVOO minority fraction, and prevalently the polyphenols, are the principal responsible for the reported healthy effects [78,79,80]. The EUROLIVE study, a randomized crossover trial in healthy volunteers, was the first study that in 2006 tested the effect of the daily 3-week administration of 25 mL of olive oils with low, medium, and high phenolic content, demonstrating the beneficial effects of phenolic compounds on oxidative damage on lipids and HDL cholesterol of high phenolic oil [81].

**Figure 2:**
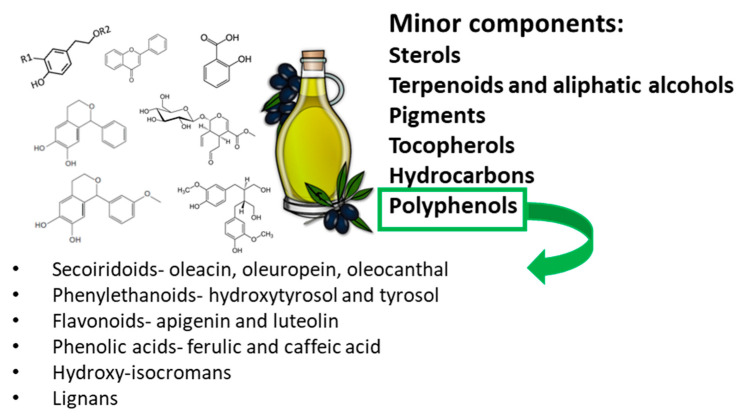
EVOO minor components—list of polyphenols.

Polyphenols include phenolic alcohols such as tyrosol and hydroxytyrosol, secoiridoid derivatives, lignans, and flavonoids. The secoiridoid derivatives, in turn, include oleuropein aglycon (present only in leaves and olive fruit) and oleacein as derivatives of hydroxytyrosol, and oleocanthal and ligstroside aglycone as derivatives of tyrosol (Figure 2).

They reduce oxidative stress and are well-known to perform a vasoprotective, anti-inflammatory, anticoagulant, and antitumor action [82].

The quality and the concentration of polyphenolic species of olive oil depend on both genetic and agronomic factors [83]. The olive cultivar, the geographical growing area (including soil composition, and the climate) as well as the agronomic practices (i.e., fertilization, and water availability) play a pivotal role in the production of polyphenols [84]. In addition, very important is the olive oil extraction process: parameters such as temperature and oxygen level greatly influence the presence of polyphenols in the final oil [85,86,87,88].

Currently, there is wide scientific literature on the bioactivity of olive oil polyphenols that underline the anti-oxidant action of oleocanthal, oleacein, oleuropein, and hydroxytyrosol. In detail, the phenolic components inhibit the process of carcinogenesis both through anti-oxidant and anti-inflammatory actions and by interfering with specific cellular mechanisms. Moreover, these molecules decrease the risk of the development of diabetes, cardiovascular, and chronic degenerative diseases [89,90,91,92].

**Figure 3:**
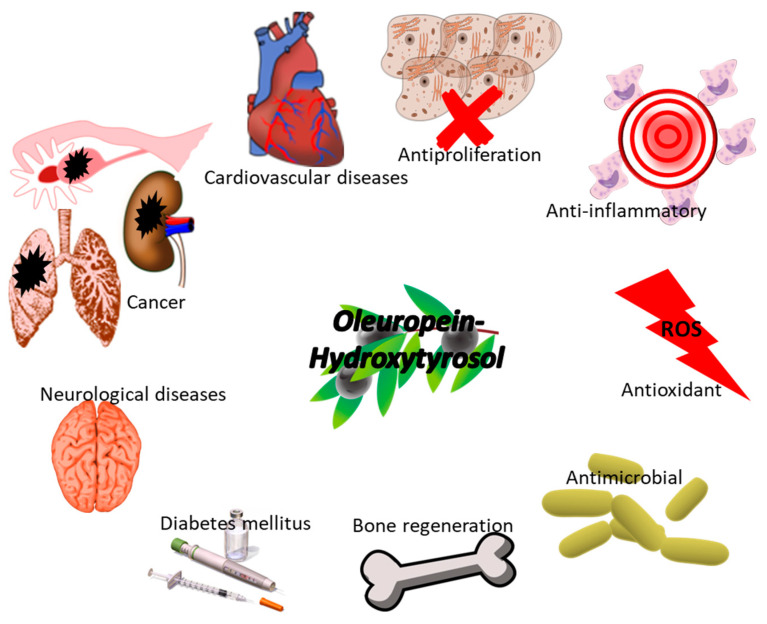
Biological activity of OLE and Htyr for human wellness.

The main beneficial health effect of oleuropein (OLE) and hydroxytyrosol (Htyr) are summarized in Figure 3.

The European Food Safety Authority (EFSA) has declared a health claim about the involvement of olive oil polyphenols in protecting LDL from oxidation: “A daily intake of 20 g of olive oil, which contains at least 5 mg of hydroxytyrosol and its derivatives (e.g., oleuropein and tyrosol) provides the expected beneficial effects”. In particular, hydroxytyrosol metabolites (Htyr-sulfate as well as homovanillic acid sulfate and glucuronate) are able to bind high-density lipoprotein (HDL), preventing their oxidative modifications. Thus, these metabolites reduced coronary event incidence and the development of early atherosclerosis [89].

The antioxidant potential of Htyr depends on both the presence of the o-dihydroxyphenyl component that operates as the chain breaker and the induction of phase II detoxifying enzymes through the activation of the nuclear factor E2-related factor 2 (Nrf2) in several tissues. Moreover, Htyr shows an anti-inflammatory effect, influencing the expression of pro-inflammatory agents such as inducible nitric oxide synthase (iNOS), cyclooxygenase-2 (COX-2), tumor necrosis factor-α (TNF-α), and interleukin (IL)-1β. The anti-inflammatory activity is also related to the prevention of granulocyte and monocyte activation. In addition, it was demonstrated that olive oil phenolics, and in particular, Htyr, are involved in epigenetic regulation such as miRNAs expression, DNA methylation, and histone modifications [89,93,94].

Due to the presence of multiple hydroxyl groups in its structure, Htyr is very sensitive to air and light. This characteristic induces a very strong instability and hydrophilicity of Htyr, influencing its biological activity. Moreover, the chemical features of Htyr make this molecule inappropriate to enrich foods or in use for several drug formulations. For these reasons, encapsulation strategies have been developed and applied to successfully deliver Htyr. Usually, solid particle systems are synthesized using solid lipids such as triacylglycerols or waxes, while polymeric ones are built of natural polymers such as chitosan, or synthetic such as polylactide co-glycolic acid (PLGA). Liposomes, small spherical vesicles consisting of one or more bilayers of phospholipids, are usually formed by cholesterol and/or triglycerides for Htyr delivery [48].

Hussain et al. [50] synthesized self-assembled chitosan nanoparticles for percutaneous co-delivery of hydrocortisone/hydroxytyrosol useful to alleviate the signs and symptoms of atopic dermatitis. The choice of chitosan as a polymeric base depends on its mucoadhesive properties and capability in transepidermal penetration through disruption of intercellular tight junctions. Poly(lactide-co-glycolic acid-co-acrylic acid) (PLGA-co-PAA) nanoparticles loaded with Htyr and doxorubicin have also been synthesized for colon cancer therapy [52]. The in vitro results on human colon cancer cells demonstrated that these nanoparticles efficiently inhibited the expression of genes related to cancer progression, so they could represent a useful system for chemotherapy.

Oleuropein (OLE), the most abundant polyphenol in olive oil, cannot be employed to enrich food for its poor water solubility and very low palatability. The encapsulation strategy represents a practical solution to preserve this molecule from environmental damage and hide its distaste properties [41].

Recently, the elite choice for OLE encapsulation is represented by lipid nanostructured carriers (NLCs), consisting in a lipid core of solid and liquid lipids stabilized by surfactants, which guarantee a broad molecule specificity (can load both lipophilic and hydrophilic drugs), scale-up workability, stability, high drug encapsulation, controlled and targeted release, biocompatibility, and biodegradability [95]. Soleimanifard et al. [41] encapsulated oleuropein in NCLs made of different components (lecithin, linoleic acid, glycerol monostearate, soybean oil, and Tween 80) by hot high-shear homogenization accompanied with ultrasonication techniques. The setup protocol allows for the synthesis of an optimal oleuropein-loaded NLC that was more efficient and stable (any oleuropein leakage was observed in 90 days at −18, 6, and 25 °C in aqueous suspension) compared with the oleuropein-free nanocarrier and pure materials. In addition, Huguet-Casquero et al. [42] synthesized NCLs to investigate a new pulmonary formulation against lung epithelial oxidative damage. Their lipid matrix consisted of olive oil and Precirol (glycerol distearate), and showed an oleuropein encapsulation efficiency of 99% with an enhanced antioxidant potential of this polyphenol. Moreover, the biocompatibility of NLCs was demonstrated in three lung epithelial cell lines, confirming their safety for lung administration.

Another vehicle useful to deliver OLE is soybean lecithin, as reported Al-Karaki et al. [45], as a colon cancer agent. Furthermore, Muzzalupo et al. [46] successfully encapsulated olive leaf extracts, composed principally in oleuropein, into chitosan nanoparticles (CS NPs). In comparison to pure leaf extracts and commercial OLE, the leaf extract/CS NPs showed a very good efficacy against *Fusarium proliferatum*, demonstrating that this strategy could be successfully applied to reduce the dosage of fungicides potentially harmful to human health.

## 3. Wine

The Mediterranean diet is characterized by two fluid foods: olive oil and wine, mainly red. A regular and moderate wine consumption (i.e., 1–2 drinks/day or ~150–300 mL/day) was approved by PREDIMED Mediterranean Diet, since it was reported that it prevents cardiovascular diseases [96,97]. However, wine drinking should be carried out in moderation, or is forbidden by some populations for ethnic or religious reasons.

Wine is classified into red, white, and rosé, according to the type (or blend) of grape varieties, sweetness, alcohol and carbon dioxide content, vintage year, winemaking techniques, or geographic origin [98].

Different fermentation procedures and substrates (musts, skins or seeds) between red and white wine affect the final polyphenolic concentration. Red wine is known to contain 10-fold more phenolic compounds than white wine (1.8 g/L with respect to 0.2–0.3 g/L of total polyphenols) [97]. In detail, red wine contains high concentrations of polyphenolic compounds such as flavonoids (catechin, epicatechin, quercetin, anthocyanins, and procyanidins), resveratrol (3,5,4′-trihydroxystilbene), and polymeric tannins [99].

Chalons et al. [100] described the anti-inflammation potential of red wine extract (RWE) due to its capability in modulating IL-1β secretion and the NLRP3 inflammasome pathway. Inflammation could be a triggering factor for cardiovascular disease, autoimmune disorders, eye diseases, age-related diseases, and finally, cancers. Therefore, RWE could represent a strategy to prevent these inflammation-related pathologies.

**Figure 4:**
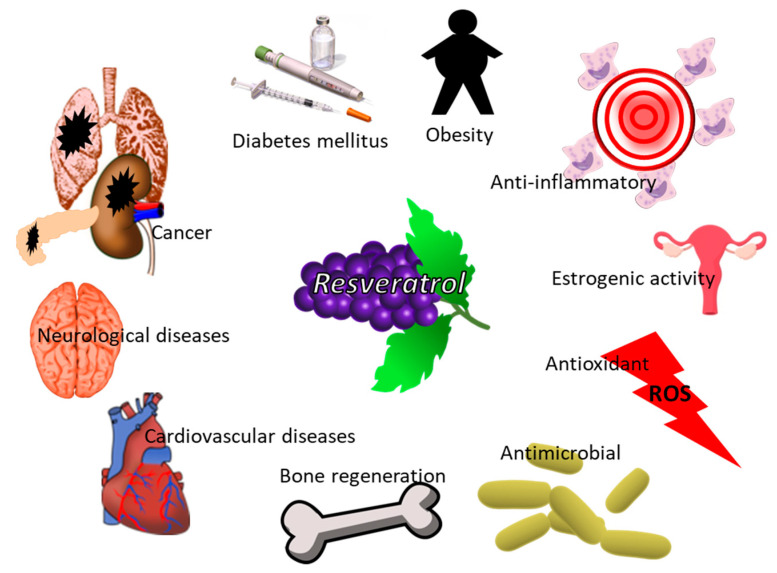
Biological activity of RSV for human wellness.

Among the wine phytochemicals, resveratrol (RSV) is one of the most widely studied, showing a plethora of healthy effects (Figure 4).

RSV sources are not only grape skins, but also berries, peanuts, and cocoa and it is synthesized to protect the plant from the attack of bacteria and fungi. There are two resveratrol isomers, cis- (Z) and trans- (E), and both have been detected in wine at concentrations ranging from 0.1 to 7.0 mg/L and from 0.7 to 6.5 mg/L, respectively [96]. Resveratrol bioactivity was related to antioxidant, neuroprotective, anti-inflammatory and anti-obesity effects and to its influence in controlling blood glucose and cardiovascular complications [101]. Moreover, it was demonstrated that dental material loaded with RSV represented a promising solution for the prevention of implant-associated infections, since the developed biomaterial was able to affect the colonization of *Pseudomonas aeruginosa* and *Streptococcus mutans*, bacteria responsible for both acute and chronic infections in humans [102].

The encapsulation strategy was also used to improve the bioactivity of resveratrol, enhancing its bioavailability. To date, numerous RSV-loaded nanoparticles (RSV-NPs) with different physicochemical properties (i.e., particle size, shape) were developed and administered in different in vivo cancer models [103,104]. A markedly enhanced anticancer activity was demonstrated, making the RSV-NPs an interesting opportunity for cancer therapy, despite further clinical trials still being necessary to allow for RSV-NPs’ clinical translation.

In particular, a variety of natural biomaterials have been investigated for their potential to encapsulate, protect, and deliver RSV. Among them, natural polymers are useful to develop enriched food or functional food. For example, zein, the major storage protein in corn, is a thermoplastic material characterized by hydrophobic/hydrophilic character, film/fiber forming, and antioxidant properties, allowing for its use in food and nutrition applications [105]. Colloidal Zein-SHA (low-molecular-weight sodium hyaluronate) nanoparticles were designed to investigate the antioxidant and antiproliferative activity in vitro of encapsulated RSV versus the free molecule. The ameliorated bioactivity obtained by Zein-SHA/RSV NPs suggested that this vehicle could be considered to create functional foods and beverages as well as dietary supplements and pharmaceutical products [55]. In another study, zein nanoparticles were coated with chitosan (CS) as a RSV delivery system. These nanoparticles exhibited antioxidant activity, thanks to the additive effect of resveratrol plus the amino acids with a zein structure. Again, the CS coating allowed for both mucoadhesive properties and a biphasic and prolonged release of resveratrol in simulated gastrointestinal fluids. For that reason, the developed system could be useful for oral administration of RSV [54].

Recently, low molecular weight co-polymer mPEG-PLA NPs conjugated with RSV were developed to evaluate the reduction in plasma degradation, hepatic metabolism, and its anticancer activity in vitro and in vivo. Study results indicated that the chemical conjugation resveratrol/NPs with respect to simple encapsulation is a valid approach to reduce the degradation and metabolism rate of RSV and consequently, increased the antitumor activity of RSV in the 57 BL/6J mouse model with melanoma [56].

Finally, several studies have reported the possibility of using RSV as an adjuvant in COVID-19 treatment [106,107]. Rossi et al. [107] reported that the already commercialized nasal spray containing carboxymethylated (1,3/1,6)-β-D-glucan (CM-glucan) combined with resveratrol could be used for COVID-19 treatment in the early phase of the infection. The CM-glucan-RSV formulation was developed in 2014 by Francioso et al. [108,109] and was demonstrated to greatly improve the severity of upper respiratory tract infections in infants with acute respiratory tract infection. It is necessary to perform randomized double-blind controlled clinical trials to validate the efficacy of this system in COVID-19 treatment.

## 4. Nuts

Nuts (tree nuts and peanuts) are an important component of the Mediterranean diet, being consumed as snacks, desserts, or part of a meal (fresh or roasted in salad), in spreads (almond paste), as oils, or hidden in commercial products, mixed dishes, sauces, pastries, ice creams, and baked goods [110,111]. Moreover, the consumption of nuts in many Mediterranean areas is increased during holiday seasons such as Christmas, when almond-based desserts such as nougat and marzipan are eaten.

Nuts contain high amounts of unsaturated fatty acids, proteins, dietary fiber, vitamins (i.e., folic acid, niacin, tocopherols, and vitamin B6), minerals (i.e., calcium, magnesium, potassium), and many other bioactive molecules, mainly phytosterols and polyphenols [111]. All of these components contribute to the positive health effects of nuts [112,113]. In particular, the antioxidant activity of nuts depends on the high content of both hydroxybenzoic and hydroxycinnamic acids including *p*-hydroxybenzoic, protocatechuic, vanillic, and syringic acids [114]. In detail, *p*-hydroxybenzoic and chlorogenic, *p*-coumaric, and gallic acids are the most abundant phenolic acids found in the kernel and skin of seeds of nut [115]. Among the polyphenols, these acids, together with ellagic acid, resveratrol, catechin, epicatechin, and quercetin, are the bioactive nut compounds most studied in the scientific world (Table 3) [116].

**Figure 5:**
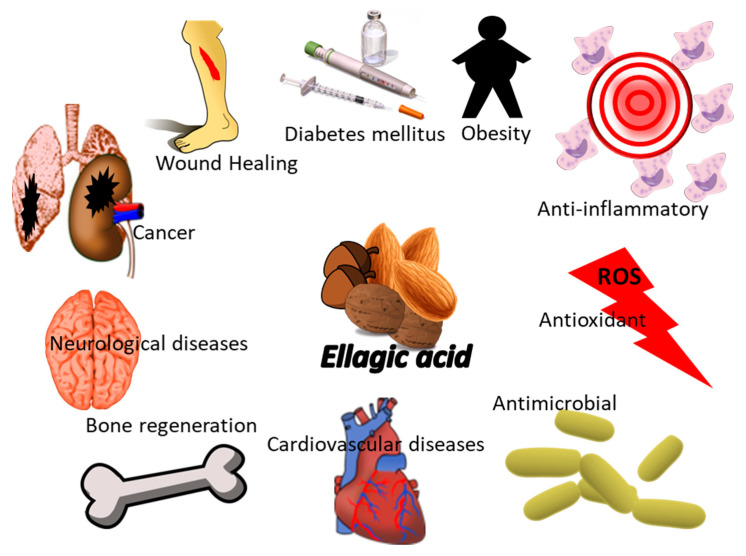
Biological activity of EA for human wellness.

In general, recent literature has reported the dietary polyphenols’ synergistic action in combination with approved drugs including anticancer to enhance their efficacy (Figure 5) [129].

In this context, pH-responsive nano-micelles (QT-CA-CS) based on chitosan, quercetin, and citraconic anhydride were investigated as a vehicle for anticancer drug doxorubicin (DOX) in multidrug resistance related tumor therapy [130]. Study results demonstrated that QT-CA-CS-DOX nano-micelles enhanced the cellular uptake of doxorubicin by drug resistance cell line MCF-7 through the escape from lysosome degradation. Furthermore, DOX in combination with released QT enhanced the inhibitory effect on this tumor cell line.

A nut polyphenol that has also been largely investigated for its healthy properties is ellagic acid (EA). EA derives from the ellagitannins (ET) family, whose molecules exhibit hexahydroxydyphenic acid residues that are subject to the esterification reaction with glucose or quinic acid [131]. EA not only shows anticancer activity but also anti-inflammatory capability, related to the inhibition of inflammatory mediator molecules such as cyclooxygenase and nuclear factor kB [132]. Interestingly, it was reported that among the nuts consumed in the Mediterranean area, almond, walnut, and hazelnut contain noteworthy amounts of ellagic acid [133]. Unfortunately, it is well-known that EA is characterized by a low bioavailability according to the most common polyphenols. Nanoliposome encapsulation could solve this condition, enhancing EA solubility and preserving it from environmental conditions, oxidation, pH change, and enzymatic degradation. In vivo experiments have reported that encapsulation of EA into phospholipid nanoliposomes improves its biological effect in preventing cyclophosphamide-induced liver damage, increasing its hepatoprotective potential [134]. Additionally, polymeric micro- and nano-systems offer protection to EA from biodegradation. In particular, biodegradable polymers, synthetic or natural, were preferred. Among the synthetic polymers, PLGA and polycaprolactone (PCL) have been largely applied since they promote EA long circulation and a sustained release over one week, while the biopolymer chitosan exhibits a more rapid EA release (50% in 8 h) [131]. Polymeric nanoparticle loading EA was studied in the hepatocellular carcinoma (HepG2) cell line for cancer treatment [57]. The authors functionalized PLGA nanoparticles with CS and PEG to deliver EA delivery in HepG2. These modified surfaces protect PLGA nanoparticles from phagocytic opsonization, prolonging their shelf life, potentiating the apoptosis-mediated cell death in HepG2 cell line.

A recent study highlighted how a micellar system can be useful to improve the bioavailability of EA, allowing it to carry out its biological effects [60]. The chosen micellar deliveries based on D-α tocopheryl glycol succinate (TPGS) were synthesized by the thin-film hydration method. TPGS was selected for its P-glycoprotein (Pgp) inhibitor capacity, a protein responsible to pour out the chemotherapeutic molecules from the cancer cells. The results of the in vitro experiments in ovarian cancer cell lines showed that encapsulated EA induced the highest cytotoxicity with respect to free EA and TPGS in a dose-dependent manner. Moreover, EA-TPGS micelles promoted the suppression of the G1/M phase through the inhibition of p15 and p21 promoter genes, related to cellular apoptotic potential.

Again, nut polyphenol content is involved in the prevention of diseases related to free radical overproduction, not only as cancer, but also as cardiovascular diseases. Pistachios are nuts very rich in polyphenols. In detail, pistachios contain anthocyanins, flavonoids, proanthocyanins, and stilbenes [135], with the flavanol catechin (CH) the most representative polyphenol (3.50 mg/100 g) [136,137,138].

Fregapane et al. [61] developed oils enriched with phenolic extracts obtained from pistachio and walnut to improve the health benefits of oil. The bioavailability of CH was also improved using PLGA nanoparticles but synthesized by adding didodecyldimethyl ammonium (DMAB) as a stabilizer and SMB (sodium metaborate) as a chelator [139]. In a serum study, it was demonstrated that the shelf life of CH could be increased with this system delivery compared to free drug. Moreover, its efficacy in the release of CH was tested in buffer phosphate medium. The results showed a continuous release profile over time (up to 70 h), making this vehicle suitable for drug delivery. A modified synthesis protocol was also applied for the preparation of chitosan nanoparticles used for the delivery of CH. Liu et al. [62] used folate-modified chitosan as a raw material to produce their nanoparticle system (CFC-NPs). The choice of folic acid depends on its capability in targeting the cell membrane and to allow the endocytosis of nanoparticles in the cancer cells, which are characterized by a significant upregulation of folate receptors. In vitro studies in human breast adenocarcinoma MCF-7 cells and hepatocellular HepG2 cells showed that CFC-NPs loaded with CH exhibited significant anti-proliferative effects in a dose-dependent manner.

Moreover, the recent need to counteract the Coronavirus pandemic situation has also encouraged scientists to explore the potentiality of natural molecules as natural anti-COVID-19 drugs. Based on in silico studies, it was hypothesized that several polyphenol and flavanol molecules could bind with high affinity to the SARS-CoV-2 spike protein and helicase, thus inhibiting the viral entry of coronaviruses [140,141].

Abou Aitah et al. [142] developed an inorganic-organic hybrid nanoformulation, composed of zinc oxide NPs (ZnO NPs), functionalized with triptycene organic molecules (TRP as 3D organic molecules) and loaded with EA to inhibit the RNA viruses H1N1 and HCoV-229E as well as DNA viruses. The produced nanoformulation was significantly more efficient as an antiviral agent than the single components when tested separately, thus demonstrating its potential as an alternative therapeutic strategy for COVID-19. However, it is necessary to explore both in vitro drug release and in vivo antiviral mechanisms before practical application as an anti-COVID-19 agent.

## 5. Spice and Herbs

Mediterranean cooking often uses spices to emphasize food taste. Spices are the dried aromatic parts of the plants [143]. The most common Mediterranean spices are basil (*Ocimum basilicum*), oregano (*Origanum vulgare*), and rosemary (*Rosmarinus officinalis*). The Food and Drug Administration (FDA) defines spices as “aromatic vegetable substances used as seasoning rather than nutrition” [144].

However, due to their high phenolic content, spices and herbs show antioxidant, anti-inflammatory, and anti-mutagen properties. For this reason, they could be useful in preventing CVD, cancer, and degenerative diseases related to the oxidative stress. Moreover, the addition of spice and herbs in food reduces the need for salt, helping in the control of blood pressure [145].

Basil and oregano have been widely used in folk medicine for the treatment of headaches, coughs, inflammation-related diseases, rheumatism, and gastrointestinal and kidney disorders [144,146]. Furthermore, their essential oils contain antimicrobial and antifungal compounds. Linalool is the main constituent of the essential oil of *O. basilicum* (28.6–60.6%), followed by estragole, methyl cinnamate, epi-α-cadinol, α-bergamotene, γ-cadinene (3.3–5.4%), germacrene D (1.1–3.3%), and camphor (1.1–3.1%). Other bioactive constituents such as myrcene, pinene, terpineol, 1,8-cineole, eugenol, and methyl-eugenol have been identified in their leaves [144]. The polyphenolic part of basil is composed of caffeic, vanillic, rosmarinic acids, quercetin, rutin, apigenin, chlorogenic, and *p*-hydroxybenzoic [147].

Meanwhile, oregano contains flavonoids, phenolic acids, and hydroxycinnamic acids (non-flavonoid phenolic). Its main flavonoid and phenolic acids are rosmarinic acid, apigenin, luteolin, quercetin, scutellarein, and their derivatives [146].

**Figure 6:**
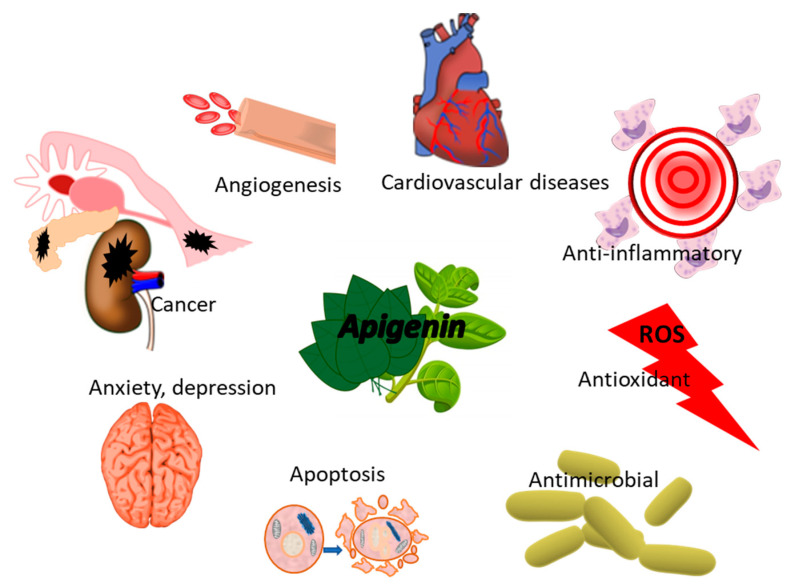
Biological activity of apigenin for human wellness.

Figure 6 summarizes the most important apigenin healthy effect.

Recent studies have reported that PLGA nanoparticles successfully encapsulated apigenin (APG) as an anticancer agent [64]. Apigenin could suppress the proliferation of cancer cells, both arresting cell cycle inducing apoptosis and suppressing metastasis through downregulation of matrix metalloproteinases and the Akt signaling pathway [148]. The developed vehicles were used to delay hepatocellular carcinoma both in vitro and in vivo (mouse model) [64]. In this regard, galactose-tailored PLGA NPs loaded with APG were also synthesized for active liver targeting to enhance NPs’ cellular internalization and apoptotic potential [65]. Moreover, the surface of the PLGA-NPs was functionalized with *meso*-2,3-dimercaptosuccinic acid (DMSA) to prevent melanoma metastasis. Indeed, DMSA-conjugated APG-NPs showed more of an apoptosis effect than the APG-NPs and could accumulate in the lungs upon its oral administration [66].

Present also in nuts and pistachios, quercetin (QUE) is widely found in spices and herbs. However, the best-known QUE sources are fruit (i.e., apples and grapes) and vegetables (onions, kale, broccoli, lettuce, and tomatoes) [15] as well as in the plant parsley and *Caralluma europea,* usually used in Moroccan popular medicine [149,150]. Due to its phenolic nature, QUE also exhibits anti-inflammation and anti-oxidative stress activity. Valentino et al. [26] reported that quercetin induced osteoblast differentiation regulating the expression of TGF-β1, BMP-2, and Runx2 via activation of ERK1/2, p38, and JNK MAPKs. Furthermore, quercetin exhibits protective potential for neurodegenerative disorders such as Alzheimer’s disease and Parkinson’s disease [151]. In particular, PVP-quercetin nanoparticles have been synthetized via pulsed laser ablation (PLA) to study the involvement of this phenol in amyloid-related diseases. The authors demonstrated that their system may act on Aβ aggregation, decreasing Aβ-induced oxidative stress and Aβ-mediated cytotoxicity [69].

Interestingly, not only apigenin, but also quercetin, have demonstrated anti-cancer effects, relating to the inhibition of COX2 and NF-κB activity and the downregulation of key proteins of the apoptosis pathway [152]. Niazvand et al. [68] investigated the use of solid lipid nanoparticles for the delivery of quercetin for the treatment of breast cancer. The encapsulation strategy allowed for an improvement in the toxicity to MCF7 cells in comparison to free quercetin, with a markedly lower half-maximum inhibitory concentration. Quercetin bioactivity was also investigated as a wound healing agent [128,153]. In this context, Choudhary et al. [71] developed chitosan-based quercetin nanoparticles able to regulate the cytokines and growth factors of inflammatory and proliferative phases of wound healing, respectively.

Finally, recent literature has recommended a phospholipid delivery form of QUE (Quercetin Phytosome^®^) as a potentially useful system for an anti-COVID-19 role [154,155]. Indeed, a 3-month clinical trial on 120 subjects demonstrated that supplementation with Quercetin Phytosome^®^ (250 mg twice a day) was able to induce a protection factor of 14% more of not contracting the COVID-19 infection than that of those who had taken a placebo [156]. Of course, nevertheless, these results are encouraging, and further studies are required to confirm the efficacy of this QUE formulation as a regular prophylaxis.

## 6. Polyphenols from Food Waste

**Figure 7:**
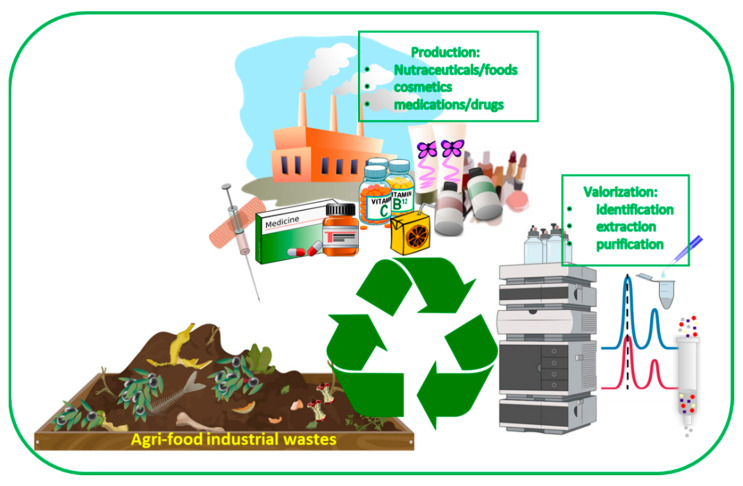
Circular economy of agri-food wastes.

The agri-food industry is the largest component of the world production market and is constantly growing. For this reason, it is necessary to find a solution for the disposal and reuse of the large amount of waste produced by food processing. The circular economy approach can solve this problem, allowing one to reduce the wastefulness of resources through the extraction and the purification of bioactive compounds, enzymes, and nutrients from industrial by-products [157]. Innovative methods such as pressured liquid, microwaves, or supercritical CO_2_ are being used for the extraction of target compounds [158]. Waste valorization started with the use of agri-food waste as a source of fuel or livestock feed or as organic fertilizers. Currently, modern technologies and the “green chemistry” concept allow for the production of added value in bioactive molecules, cosmetical and medical products, or dietary supplements by exploiting waste [158]. Moreover, recovery phytochemicals can be applied in agri-food products as antioxidants, antimicrobials, and natural pigments (Figure 7) [159].

Usually, non-invasive or room temperature treatments can be applied to preserve the secondary metabolic pool of the raw materials [160].

An example of an exploited waste product from the agro-industrial chain is the shell of nuts. In particular, pistachio hulls are largely used to develop value-added products, since they contain not only polysaccharides but several polyphenols and some of these molecules have a higher concentration in the hull compared to the nut itself [161]. Mainly, pistachio shells show important amounts of gallic acid, quercetin, cyanidin, catechin, and galloyl compounds [162]. Pistachio green hull extract (PGHE) was encapsulated in nanoliposomal carriers to protect and ameliorate PGHE bioavailability and bioactivity [163]. The reported characteristics, together with high storage stability, support the hypothesis of the use of the designed nanoliposomes in functional food production. Furthermore, grape pomace extracts (GPE) are also a rich source of polyphenols such as trans-resveratrol and other stilbenes, quercetin, proanthocyanidins, anthocyanins, hydroxybenzoic, and hydroxycinnamic acids and catechins [164]. The encapsulation of pterostilbene or non-fermented GPE in PLGA-NPs was obtained to test their antifungal activity against *Candida albicans* [165]. The entrapment method showed an efficiency of ~90%, with a higher anti-*Candida* biofilm activity than free formulations. Thus, NP encapsulation of this wine industry by-product could be a valid anti-fungal approach useful in different applications. GPE was also encapsulated into chitosan (CS) and alginate (Alg) NPs to overcome the degradation of bioactive polyphenols in the gastrointestinal tract. In vitro gastrointestinal digestion demonstrated that the two formulations enhanced the bio-accessibility of GPE phenolics and, consequently, their antioxidant and antimicrobial activities. Therefore, the developed systems could be effectively applied for oral polyphenol administrations including functional foods.

The main product of the Mediterranean area is olive oil. According to the extraction techniques used, different by-products have been developed such as the solid olive residues, representing about 35% (*w/w*) of the processed olives [166]. It is well-known that the olive cake fraction contains several polyphenols such as hydroxytyrosol, tyrosol, and the dialdehydic form of decarboxymethyl elenolic acid linked to these molecules: verbascoside, caffeic acid, *p*-coumaric acid, vanillic acid, lutein, acetoxypinoresinol, and pinoresinol. An on-farm study reported that the nutraceutical value of meat can be increased by adding olive cake in chicken feed [167]. Indeed, due to its phenolic composition, olive cake enhanced the antioxidant activity of meat without altering its quality or composition.

Moreover, among the olive oil production waste, olive leaves are the most produced by-product, followed by olive pomace, and wastewater [168]. Recently, numerous products based on bioactive molecules extracted from olive leaves have been commercialized [169]. Olive leaf extracts are largely used in food technology through the microencapsulation approach, especially as a spray dryer, using maltodextrin and trehalose as matrix components [170]. The main issues in processing olive leaves are the bitter taste, predominantly of OLE, and the low stability of phenolics. Nanotechnology could overcome these limits, improving the functionality and shelf life of olive leaf extracts [171]. The ameliorated stability of olive leaf extracts due to the encapsulation approach is also useful in cosmetics. Indeed, biodegradable nanoparticles have been used to develop a polyphenolic-enriched cosmetic formulation [172] as well as a new vehicle for olive leaf extracts to induce an antimicrobial effect [46]. Indeed, Muzzalupo et al. [46] demonstrated in vitro antifungal activity of olive leaf extracts loaded in CS NPs to control plant diseases and decrease the dosage of fungicides in the agri-food industry.

Olive leaf extract was encapsulated in a nanostructured lipid carrier and dispersed into the pectin-sodium caseinate hydrogel network to reduce the oxidation in high-fat foods and improve their nutritional properties at the same time [173]. The author reported that the olive leaf extract antioxidant activity was preserved after 40 days of storage at 4 °C, resistant to adverse environment factors.

Tavakoli et al. [174] synthetized phosphatidyl choline/cholesterol nanoliposomes loaded with olive leaf extracts to ameliorate yogurt nutritional value, taste, and properties.

Finally, according to the concept of a circular economy, food industry by-products could be reused as energy sources. Indeed, a considerable number of valorization or value-added strategies are available for substituting present feedstocks for biofuel production with organic wastes from fruits, vegetables, nuts, and cereals. These do not only ensure adequate food for human and animal feed, but further reduce the deleterious landfilling and open dumps considered in existing waste disposal methods. As such, this approach is more environmentally friendly, sustainable, and profitable [167].

## 7. Conclusions and Future Perspectives

The healthy benefits of the Mediterranean diet are predominantly associated with the presence in the foods of secondary metabolites, mainly polyphenols, whose healthy characteristics are widely recognized. The scientific research of the last decades has focused on overcoming the drawbacks of food-derived bioactive molecules such as very low bioavailability, lower absorption from the intestine, extensive gut transformation, and rapid clearance from the body. As discussed above, some of these limitations have been improved by the development and optimization of different delivery systems such as liposomes, nano- and micro-emulsions, solid lipid nanoparticles, and polymeric nanoparticles. These promising nanoformulations showed significant in vitro effects in the prevention and counteraction of numerous pathologies such as cancer, cardiovascular diseases, metabolic syndrome, and neurodegenerative diseases. However, long-term in vivo studies should be conducted to better elucidate the role and the advantages of these nanotechnological systems. In this context, the circular economy strategy based on the possibility of recovering polyphenols from food waste, meets the exigence to obtain valuable biomolecules at low cost and in high quantity to simultaneously respond to the economic and ecological exigencies of our society.

## Figures and Tables

**Table 1:** Principal MD bioactive polyphenols and their vehicles discussed.

Sources	Compounds	Delivery Systems *	Reference
Olive oil	Oleuropein	NLS	[41,42,43,44,45]
		CS NPs	[46,47]
	Hydroxytyrosol	Liposomes	[43,44,48]
		CS NPs	[26,49,50,51]
		PLGA-co-PAA NPs	[52,53]
Wine	Resveratrol	Zein-SHA and zein-CS NPs	[54,55]
		mPEG-PLA NPs	[56]
Nuts	Ellagic acid	polymeric NPs	[57,58,59]
		TPGS micelles	[60]
	Catechin	PLGA NPs	[61]
		CS NPs	[62,63]
Oregano and basil	Apigenin	PLGA NPs	[64,65,66]
		SLN	[67]
	Quercetin	SLN	[68]
		PVP	[69,70]
		CS NPs	[71,72]

* Legend: NLS = Lipid nanostructured carriers, CS = chitosan, NPs = nanoparticles, PLGA-co-PAA = Poly D-L-lactide-co-glycolic-co-acrylic acid, Zein- sodium hyaluronate = zein-SHA, mPEG = polyethylene glycol monomethyl ether, TPGS = D-α tocopheryl glycol succinate, SLN = solid lipid nanoparticles, PVP = polyvinylpyrrolidone.

**Table 2:** Nutritional components of olive oil.

	Component	Amount
Saponifiable fraction	Saturated FA	14%
	Total unsaturated FA	>85%
	Mono-unsaturated	73–75%
	Poly-unsaturated	13–15%
Unsaponifiable fraction	Phenolic compounds	50–1000 mg/Kg
	Phytosterols	300 mg/100 g
	Tocopherols	97 to 785 mg/kg
	Pigments	from few ppm up to 25 ppm

**Table 3:** Main nut polyphenols and their bioactivity.

Compound	Nut Species	Bioactivities	Reference
Ellagic acid	Almond, walnut, hazelnut	inhibitor of inflammatory mediators, anticancer, cardiovascular and neurodegenerative diseases treatment, wound-healing properties, antibacterial and antiviral effects	[117]
Gallic acid	Almond, hazelnut, pistachio, walnut	Antioxidant, anti-inflammatory (allergic inflammation), anticancer, antimicrobial, pulmonary and gastrointestinal treatment, protective effect on neuropsychological diseases	[118]
p-Hydroxy benzoic acid	Peanut, almond, walnut, hazelnut	antioxidant and antibacterial properties, anti-tumor activity	[119,120]
p-Coumaric acid	Peanut, almond, walnut, hazelnut	Antioxidant, anti-inflammatory, antimicrobial, antiviral, anticancer, hyperlipidemia and diabetic treatment	[121,122]
Catechin	Peanut, almond, pistachio, walnut, nut, hazelnut	Antioxidant, antimicrobial, antiviral, anti-inflammatory, anti-allergenic, anticancer, cardiovascular and neurodegenerative diseases treatment prevention	[123,124]
Epicatechin	Peanut, almond, pistachio, hazelnut, walnut	Antioxidant, antidiabetic, anticancer, anti-inflammatory, antihypertensive, brain disorders treatment	[125,126]
Quercetin	Peanut, almond, pistachio, hazelnut, walnut	Antioxidant, anti-inflammatory, anticancer, antibacterial, antiviral, cardiovascular disease prevention, brain disorders treatment	[127,128]

## Data Availability

Not applicable.
